# Advancements in Learning-Based Navigation Systems for Robotic Applications in MRO Hangar: Review

**DOI:** 10.3390/s24051377

**Published:** 2024-02-21

**Authors:** Ndidiamaka Adiuku, Nicolas P. Avdelidis, Gilbert Tang, Angelos Plastropoulos

**Affiliations:** 1 Integrated Vehicle Health Management Centre (IVHM), School of Aerospace, Transport and Manufacturing, Cranfield University, Bedfordshire MK43 0AL, UK; 2 Centre for Robotics and Assembly, School of Aerospace, Transport and Manufacturing (SATM), Cranfield University, Bedfordshire MK43 0AL, UK

**Keywords:** robotics, machine learning, MRO hangar, robot navigation, object detection, deep learning

## Abstract

The field of learning-based navigation for mobile robots is experiencing a surge of interest from research and industry sectors. The application of this technology for visual aircraft inspection tasks within a maintenance, repair, and overhaul (MRO) hangar necessitates efficient perception and obstacle avoidance capabilities to ensure a reliable navigation experience. The present reliance on manual labour, static processes, and outdated technologies limits operation efficiency in the inherently dynamic and increasingly complex nature of the real-world hangar environment. The challenging environment limits the practical application of conventional methods and real-time adaptability to changes. In response to these challenges, recent years research efforts have witnessed advancement with machine learning integration aimed at enhancing navigational capability in both static and dynamic scenarios. However, most of these studies have not been specific to the MRO hangar environment, but related challenges have been addressed, and applicable solutions have been developed. This paper provides a comprehensive review of learning-based strategies with an emphasis on advancements in deep learning, object detection, and the integration of multiple approaches to create hybrid systems. The review delineates the application of learning-based methodologies to real-time navigational tasks, encompassing environment perception, obstacle detection, avoidance, and path planning through the use of vision-based sensors. The concluding section addresses the prevailing challenges and prospective development directions in this domain.

## 1. Introduction

In recent years, the aviation sector has made significant strides in the periodic inspection and maintenance of aircraft, aiming to keep pace with the increasing global air traffic demand. This focus is driven by a commitment to safety and the goal to reduce operational costs, which currently represent 10–15% of airlines’ operational costs and are projected to rise from $67.6 billion in 2016 to $100.6 billion in 2026 [1]. This has heightened interest in automated visual aircraft inspection with the aim of reducing conventional assessment strategies conducted by human operators, which are often time-intensive and susceptible to transcriptional error, especially when accessing complex and hazardous areas within the aircraft [2]. To overcome these limitations and improve the effectiveness of the aircraft visual inspection process, the aerospace industry is actively exploring the integration of unmanned robotic systems, including mobile robots and drones. The fundamental focus lies on the capacity of robots to perceive and navigate through their surroundings, ensuring the avoidance of collisions with obstacles. This necessitates the understanding of dynamic and unstructured environments, like aircraft hangars, where accurate and real-time detection and avoidance of obstacles have paramount significance [3]. The hangar environment is unpredictably complex, with diverse object irregularities, including light variations that contribute to environmental uncertainties and navigational difficulties. Consequently, there is a need to equip autonomous vehicles with reliable obstacle detection and avoidance mechanisms to improve their ability to safely navigate the surrounding environment.

Traditionally, mobile robots have utilised technologies such as Radar and GPS, along with various other sensors for navigation purposes. However, in comparison to these sensors, RGBD (red, green, blue—depth) cameras and LiDAR (light detection and ranging) systems, although more expensive, offer significantly broader range and higher resolution. These advanced sensors enable the capture of a more detailed representation of the environment. RGBD cameras provide a rich visual and depth perception, while LiDAR systems offer more precise environmental mapping, making them superior for complex navigation tasks [4]. The data collected by these sensors undergo algorithmic processing to create comprehensive models of the environments that enable the implementation of obstacle avoidance strategies. The use of mobile robots to perceive, detect, navigate through environments, and enhance inspection processes has gained considerable attention in this field [5]. However, the principal challenge extensively investigated is accomplishing a navigational task that ensures an optimal, collision-free, and shortest path to the designated target. This challenge is amplified by the inherently complex and unstructured nature of the changing environments, which complicates the real-time decision-making process and impacts the robot’s autonomy. Consequently, the robots struggle to navigate, avoid obstacles, and identify the most suitable path in changing environments.

Previous studies have demonstrated various methods of autonomous navigation, with significant attention given to conventional approaches that combine local and global path planners. These methods, including the dynamic windows approach (DWA) [6], rapidly exploring random tree (RRT) [7], and Dijkstra [8], have shown notable results, especially in static environments, and can successfully navigate robots from one point to another with a reasonable level of confidence that they will avoid collisions with obstacles. However, these approaches frequently rest on a set of presumptions that are unlikely to be true in practice [9], involve a significant computing burden [10], and manual tuning of system parameters [5]. Also, it requires extensive engineering effort to develop and adapt the system to different environments, especially where there are dynamic obstacles of varying shapes and sizes.

Recent studies have demonstrated an increasing interest in learning-based techniques, underpinned by advancements in deep learning and computer vision, for their efficacy in self-learning, optimisation, and adapting to variable conditions. This transforms raw sensory input into an adaptive understanding of the environmental features, facilitating obstacle detection, avoidance, and path planning in unstructured and complex scenes. This equips mobile robots with the capability to tackle the intricacies of dynamic real-world environments effectively. The work in [11] was applied to learn navigation tasks and plan a safe path efficiently. Nagabandi et al. [12] addressed adaptability and generalizability in dynamic environments, while Koh et al. [13] improved real-time capabilities for effective navigation experience in aircraft inspections. While numerous mature algorithms for obstacle avoidance exist, the development of highly robust obstacle avoidance algorithms, particularly those enabling robots to operate effectively in unstructured environments, remains an area meriting further investigation.

**Figure 1:**
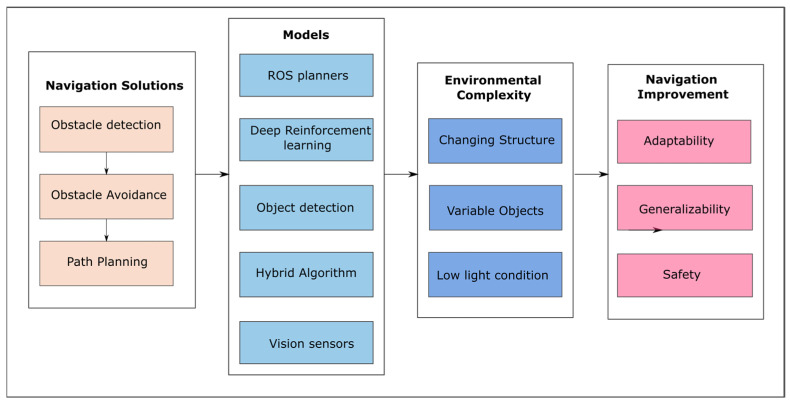
Overview of the learning-based architecture in this survey.

This research work focused on offering a comprehensive analysis of learning-based approaches applied to autonomous ground robot navigation within complex environments. It aimed to facilitate obstacle detection, obstacle avoidance, and path planning within the challenging and confined spaces of MRO hangar environments, striving for safe and intelligent navigational outcomes. The application of deep-learning-based detection techniques, including deep reinforcement learning DRL [11], Fast R-CNN (Fast region convolutional neural network) [14], and YOLO (you only look once) [15], have been extensively studied. These models exhibit a wide range of capabilities in detecting obstacles with varied and unpredictable forms, dimensions, and even under challenging lighting conditions. Each technique employs unique methods; DRL utilises policy, value, and reward systems, while YOLO and FAST R-CNN use bounding boxes and class labels to provide comprehensive information. In combination with sensor fusion, visibility in low-light situations and improved detection capability have been shown to be attained, consequently enhancing obstacle-free path planning and adaptation to a wide range of scenarios. Multiple sensor information creates a more robust and accurate representation of the environment, which is used to train and enhance the applicability and reliability of the model [16]. This study presents numerous learning-based mobile robot navigation algorithms that have been proposed to address navigation difficulties, enhance motion decisions, and generalise in changing and complex hangar scenes. These methodologies are organised into three distinct categories, deep learning, object detection and hybrid approach, all with a focus on improvements in environmental perception, adaptability, and safe navigation capabilities in handling complexities and uncertainties inherent in a changing and dynamic environment. The core components of learning-based methods discussed in this review work are shown in Figure 1. The major contributions are summed up as follows:

- This study examined learning-based navigation strategies, emphasising efficiency, safety, and adaptability for use in complex settings such as MRO hangars.
- The review categorised algorithms based on deep learning, obstacle detection, and hybrid techniques, contributing to the generation of optimal and safe paths in a given environment.
- It elucidates the challenges faced by these algorithms and potential directions for their applications in real-world scenarios.

The rest of the sections are structured as follows: Section 1 highlights some related literature. Section 2 presents the concept of machine learning and robotics as industry 4.0 (I4.0) technologies with their significance in autonomous mobile robot (AMR) systems for the digitalisation of aircraft inspection and maintenance operations. Section 3 presents the learning-based frameworks and potential use cases in aircraft repair maintenance and repair operations. In Section 4, we discuss associated difficulties, future trends, and opportunities, critically looking at simulation to real-world transfer, and finally, the conclusion is in Section 5.

## 2. Related Work

In recent years, there has been remarkable advancement in the field of obstacle detection, avoidance, and path planning in different domains. This advancement has played a significant role towards realising the vision of the “Hangar of the future”, a concept that envisions a highly automated, efficient, and safe environment for aircraft maintenance and inspection. Recent studies have engaged AMR for navigation and safe interaction with its environment to automate aircraft inspection in a hangar environment [17]. Thanavin et al. [18] investigated robot navigation mechanisms for aircraft inspection in complex hangar environments. In [19], the authors proposed an automated approach to aircraft inspection using a depth camera-based mobile robot by following a predefined path to the target location. Another work applied obstacle avoidance control with the mobile robot while navigating around an aircraft for visual inspection [20]. However, much research has not been conducted specifically on hangar environments related to obstacle detection, avoidance, and path planning strategies, but similar challenges have been addressed in recent research studies. This harnesses the power of deep learning techniques and the capabilities of various visual sensors, such as cameras, LiDAR, and depth cameras, to improve the navigational abilities of mobile robots. This primarily focuses on overcoming the limitations of traditional methods by enhancing environmental perception and adaptability mechanisms, thereby effectively addressing unforeseen complexities and uncertainties in environmental factors [11]. Many extensive reviews have been conducted to delve into the application of learning techniques in mobile robot navigation, encompassing aspects like obstacle detection, obstacle avoidance, and path planning.

### 2.1. Navigation with Object Detection Model

Deep learning has emerged as a promising technique for solving object detection problems and challenges. Kuutti et al. [21] conducted a comprehensive survey highlighting the multifaceted capabilities of deep learning. These include managing multimodal sensor data, extracting features, learning complex and high-dimensional states, and addressing advanced object recognition challenges. In [22], the authors reviewed recent and successful object detection methods that have significantly advanced the field of autonomous vehicles. Furthermore, the study in [23] provided an overview of object detection and segmentation systems specific to autonomous vehicles, focusing on the detection methods, sensors, and fusion capabilities to achieve results and extend to related challenges in other application domains. The research paper in [24] delved into the range of object detection and tracking techniques, emphasising their generalizability in complex settings. The works by Gupta et al. [22] discuss the capabilities of object detection models through the evaluation of object detection metrics from sensor input. The implementation of the YOLOv5 object detection model was introduced in [25], which demonstrates reduced computational demand and improved system accuracy when tested on KITTI datasets. However, a limitation noted in the review study [26] was its ineffectiveness in detecting certain environmental objects. The application of the Faster R-CNN two-stage object detector model, as discussed in [27], addresses detection issues by providing efficient learning-based object detection and tracking solutions for autonomous vehicles. This model, however, faces challenges due to its structural complexity and high computational demands.

### 2.2. Navigation with Deep Learning Model

The application of deep reinforcement learning represents another facet of deep learning methods, specifically in addressing challenges related to robot obstacle avoidance and path planning [28]. Diverse modalities have been analysed, including learning from scratch in both model-based and model-free states, as well as learning from experience. The authors in [25] reviewed the transition from classic methods of obstacle avoidance and motion planning to the capability of learning-based approaches, yielding notable results. However, this review focused on the application of RL only in mobile robot autonomous navigation. Major limitations of DRL in real-world service robots were elaborated in [29], and to address these challenges, Zhao et al. [30] explored DRL methods that represent a synergistic combination of RL and DL. They highlighted the significant contributions of DRL in achieving optimal path planning and efficient navigation, particularly in the context of constraint factors. Shabbir et al. [31] reviewed the capabilities of deep learning through environmental perception and modelling for an efficient navigation experience. Most models integrate Q-learning techniques to solve navigation task challenges through path planning and obstacle avoidance using discreet actions [32,33]. The authors in [34] used a reward function and continuous action space to achieve safe navigation tasks. Imitation learning, which is the act of behaviour cloning, has also been adopted recently by many scholars with outstanding success. Abdelwahed et al. [35] demonstrated the use of experience solutions to learn and solve new problems using machine learning concepts. In contrast, the one by [36] reviewed imitation and reinforcement learning methods that build essential obstacle perception and control mechanisms from environmental data for the fully autonomous and intelligent robot navigation system.

In another study, the application of behaviour-cloning techniques using the fuzzy controller [37] and from human demonstration [38,39] demonstrated optimisation of the robot’s navigation experience. The concepts of intelligent robot navigation have been significantly enhanced with learning-based systems, including supervised learning that focuses on feature extraction and adaptability in changing environments leveraging vast amounts of data [40]. Some of these have been integrated into conventional algorithms to enhance their suitability in real-time applications and real-world environments.

### 2.3. Navigation with Hybrid Model

The combination of classical path planning and learning techniques, which is presumed to offer greater practical stability, has garnered considerable attention. A comprehensive review of navigation conditions in static and dynamic environments was conducted by Patle et al. [41], exploring potential hybrid mobile robot navigation techniques and the suitability of their performance across various environments. In their work, Deshpande et al. [42] explored the advantages of hybrid methods with reference to different areas of application. Janji et al. [43] conducted a review focusing on the integration of neural-network-based solutions, giving attention to input, output, and environment state, as well as their ability to address major obstacle avoidance and path planning constraints. The work in [44] comparatively analysed heuristic neural network algorithms for path planning and obstacle avoidance. Du et al. [45] further explored the application of real-time neural network algorithms for generating collision-free routes to the destination, aiming to improve the motion control and obstacle detection accuracy of robot systems. Overall, this paper leveraged many learning-based navigation method studies, providing a broader understanding and recommendation on the application and suitability of recent models based on their functionalities in complex environments.

## 3. Concept and Background

### 3.1. MRO Hangar in Aviation

**Figure 2:**
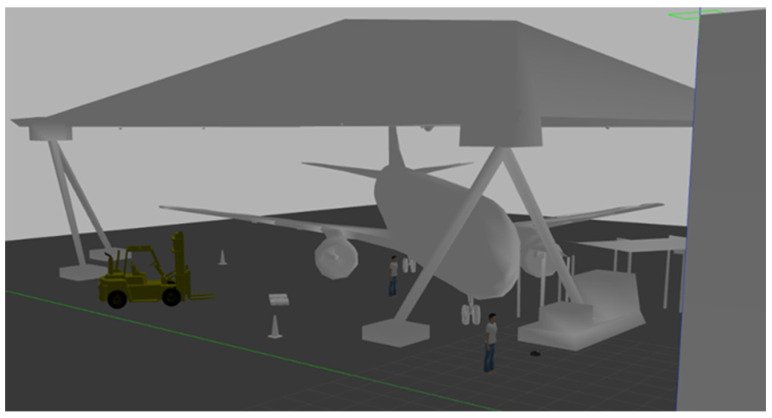
Simulated Cranfield University MRO hangar.

**Figure 3:**
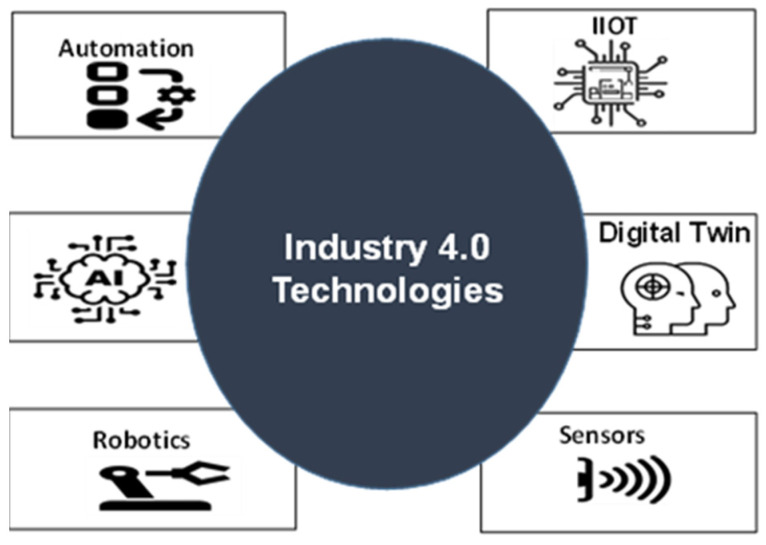
Industry 4.0 technologies.

The aerospace industry has continually evolved to guarantee the safety and reliability of aircraft to make air travel one of the safest and most reliable means of transportation. The traditional approach to aircraft maintenance and inspection involves semiautomated systems with human control to execute tasks. Sensing and navigation systems are usually preprogrammed to follow predefined inspection paths and do not adapt to unexpected conditions or obstacles. These factors are time-consuming and increase the overall operation cost. There is a growing need for more advanced and automated systems, potentially reducing cost and enhancing safety. The aviation industry has grasped the integration of robotics to improve the MRO processes of aircraft towards the “Hangar of the Future” initiative. The MRO hangar represented in a simulation model shown in Figure 2 is a major part of the aviation sector in which Industry 4.0 (I4.0) technology environments [17] have gained wide adoption and are instrumental in improving safety and operational efficiency in the environment. Robotics and artificial intelligence are part of the key enablers of I4.0, as illustrated in Figure 3. These technologies have been effectively harnessed using unmanned vehicles, including intelligent ground robots, for autonomous navigation in a busy and changing hangar environment, particularly for inspection, maintenance, and repair tasks. A comparative description of the intelligent application of robotics over the conventional method is shown in Table 1. Robots have emerged as a promising cutting-edge technology, enabling efficient and precise operations in various tasks, including assembly, drilling, painting, and inspections. Intelligent robots involve the use of machines that are built and programmed to perform a specific task, combined with artificial intelligence techniques that instil and optimise intelligence through automated, data-driven learning capabilities. The integration of these technologies has spurred a digitalisation drive within the sector, promoting the concept of “Hangar of the future”. This is where intelligent robots play major roles by improving aircraft inspection efficiency and reducing aircraft-on-ground (AOG) time and overall operation cost.

A typical hangar environment is characterised by highly complex configuration space due to the presence of unstructured and dynamic objects that vary in shape, size, and colour. Additionally, low-light conditions prevalent in such environments can impact visibility in certain areas. These factors pose a challenge for robots, as their ability to navigate from the starting point to the target location is constrained by objects and changing environmental structures [46]. Mobile robots must proactively engage with their surroundings, interacting with and exploring the aircraft environment to ensure efficient navigation experiences [17]. In this process, they generate valuable information using various sensors that facilitate the detection of environmental features, including positions of obstacles, which are essential for environment modelling and safe navigation to their destinations [47]. Different machine-learning-based functionalities have been developed, leveraging environmental information for robotic applications [28]. These have been demonstrated through various robotic platforms, such as the human-like robots from Boston Dynamics, the Crawling inspection robot by Cranfield University [2], and others. These robot systems follow standard robot architecture comprising sensory data acquisition, environmental perception, decision-making process, and execution of actions. This architecture is embedded within the robot’s hardware framework to effectively learn the robot’s orientation relative to a set of state space variables for optimal navigation in complex and dynamic environments.

### 3.2. Intelligent Robotic in MRO Hangar

**Figure 4:**
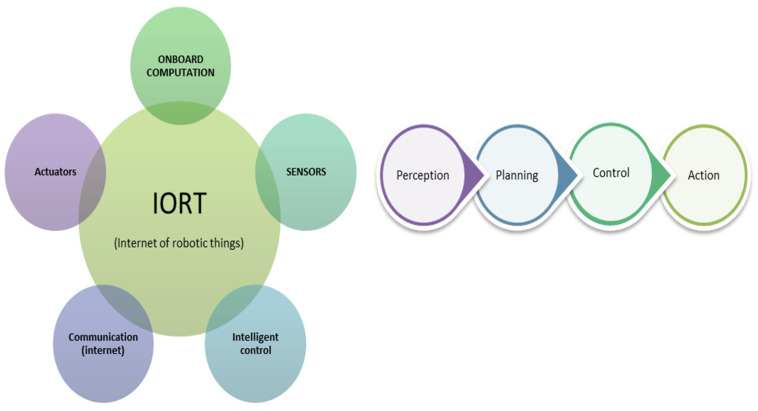
Structure of robotic intelligence.

Mobile robots encompass comprehensive system structures that work together through perception, detection, motion planning, and control, as illustrated in Figure 4, to perform a series of navigation tasks. Robotic scientists in this field have proposed many intelligent technologies integrated to form an Internet of robotic things (IORT) that can interact with the environment and learn from sensor information or real-time observation without the need for human intervention [2]. This technology empowers robots to operate more independently and make decisions based on the information they gather from their environments. Machine learning (ML) is an artificial intelligence approach that is at the core of these enabling intelligent technologies with widespread adoption [2] and has become an essential component in accomplishing many intelligent tasks in robotics. The ML techniques incorporate sensor information fusion, object detection [48], collision avoidance mechanism [49], pathfinding [50], path tracking [51], and control systems [21] to solve robot autonomous navigation problems [52]. Diverse arrays of sensors, including laser scanners, cameras, LiDAR, and others, are leveraged for information gathering, mapping, obstacle detection, as well as robot positions and velocities. The fusion of information from these multiple sensors has brought a paradigm shift in the development of more robust and accurate models of robotic systems. The multisensory fusion augments the capabilities of each individual sensor, thereby enhancing the overall system’s visual perception and its efficacy in obstacle detection and avoidance under a variety of operational conditions [53]. ML methods have revolutionised robot navigation, especially in unstructured and complex environments, by offering highly accurate and robust capabilities [54] to training models by learning from data to adapt to various types of obstacles they encounter during navigation.

### 3.3. Robotic Navigation

**Figure 5:**
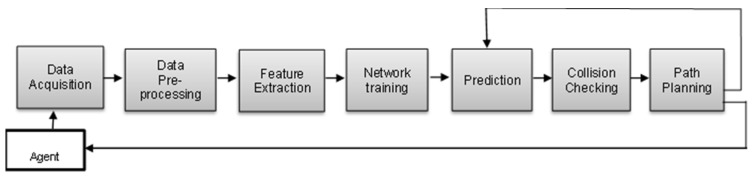
Learning-based path planning framework.

The subject of autonomous robot navigation entails mapping, localisation, obstacle detection, avoidance, and achieving an optimal path from a starting point to a predefined target location efficiently [55]. The nature of obstacles encountered can be static or dynamic, depending on the environment structure. Navigation through such an environment can be challenging due to the reliance on the sensory and real-time capability of analysing the vast amount of environmental data. Some of the robot navigation problems include the need to accurately perceive, identify, and respond to the geometry of the environment, the shape of the robot, obstacle types, and obstacle position using a suitable model. However, improper navigation processes often result in inaccuracies in perception, the development of flawed models of the environment, and emergencies of learning complexities, which significantly limit the robot from achieving its navigational goal. The application of advanced computational techniques like parallel processing and deep neural network (DNN) algorithms has significantly improved the navigation experience. In the context of a neural-network-enabled approach for obstacle avoidance and path planning, the architecture encompasses several interconnected modules, each contributing uniquely to overall system efficiency. As illustrated in Figure 5, the modules collaboratively contribute to achieving optimal path planning, adapting to changing scenarios, and are able to minimise obstacle collisions in complex environments.

For many applications, various researchers have added specialist knowledge and undertaken studies to improve these modules to solve navigation problems. In most cases, the agents learn from data or through trial and error to master navigational skills and facilitate the generalisation of learned skills in similar settings in simulation environments, which is valuable for reducing training time and real-world difficulties. The virtual platform helps to manage environmental factors and task structure that can influence the efficiency, adaptability, and reusability of these models before transferring to the real-world environment. In the context of the MRO hangar environment, the robotic systems are subject to complexities and uncertainties due to the unstructured nature of the settings, variability in object types, and sensor capacity. This demands robust solutions capable of perceiving, responding and adapting to real-time changes.

### 3.4. Vision Sensors

To effectively perform robotics tasks, mobile robots require a thorough understanding of their environment. To achieve this, robots are equipped with sensors that enable them to perceive and gather relevant information from the surroundings. Vision-based sensors, including LiDAR, cameras, and depth cameras, have become the most used equipment for unmanned vehicle (UV) detection and navigation tasks [56]. LiDAR is extensively used in the detection and tracking of AMR, even though it may be more costly than some alternatives. The sensor can obtain reliable information, including basic shape, precise distance measurements, and position of the obstacle, and is more efficient in different weather and lighting conditions [57]. However, the ability to capture the texture and colour of objects for accurate obstacle detection is limited compared with cameras [58,59]. This limitation can result in challenges when attempting to accurately track fast-moving objects in real time. RGBD cameras have also shown great capabilities, including high resolution and generation of rich and detailed environment information, though within a limited range, but are greatly efficient in object position estimation using depth information [60,61]. However, the performance is highly susceptible to lightning conditions, which can be associated with certain areas in the hangar environments. The hangar environment has significant influences on the choice of appropriate perception sensors for operational use within the space. Obstacle detection sensors are designed to interact with the environment and generate environmental data through sensor devices. They then use algorithms based on computer vision and object recognition for obstacle detection, tracking, and avoidance in a navigation system. To complement the capabilities of the RGB camera, depth sensing was combined in [62] to provide an accurate distance between obstacles and the robot position based on operational range and resolutions. The authors in [63] employed depth camera information to estimate robots’ poses for an efficient navigation experience. Depth cameras like Microsoft Kinect, Intel RealSense, and OAK-D offer valuable 3D spatial data that can enhance robots’ understanding of their environment with precision. The integration generally facilitates obstacle sensing and state estimation for robust obstacle avoidance and path planning. Like the RGB cameras, variable lighting conditions and environmental factors can affect the accuracy of the perceived obstacles and position. This perception constraint is part of obstacle avoidance and path planning challenges in complex settings.

Recent research has made significant contributions to intelligent obstacle detection and avoidance solutions based on sensor usages and algorithm improvement. The work in [64] presents different configurations and capabilities of vision sensors relevant across diverse domains. Manzoor et al. [65] analysed Vison sensor modalities as intricate factors in understanding environmental features used in deep learning models for real-world mobile robot’s obstacle detection and navigation operation. Xie et al. [66] improved obstacle detection and avoidance techniques through the utilisation of 3D LiDAR. Their study highlights the proficiency of LiDAR in detecting basic shapes and identifying obstacles at extended ranges. The integration of sensor data for more comprehensive environmental perception in learning-based models has been a notable development in the field of robotic navigation. This translates raw sensor data into usable information to enhance the system’s capability from environmental perception to improved efficiency in obstacle detection and effective decision making for obstacle avoidance and path planning.

### 3.5. Obstacle Detection

Obstacle perception and identification for robot navigation involves locating potential obstacles that could influence a robot’s ability to navigate in its surroundings. The mobile robot utilises its sensory systems, which may include LiDARs or cameras, to perceive and understand its environment, enabling it to plan a safe and collision-free path to its intended destination. Deep learning has gained wide adoption in research and industry, leading to the development of numerous navigation models that leverage different object detection models and sensor inputs for robot obstacle detection and avoidance systems. Most recent object detection methods are based on convolutional neural networks (CNNs) like YOLO [67], Faster R-CNN [14], and single-shot multibox detectors [68]. Faster R-CNN is renowned for its high detection accuracy and employs a two-stage deep learning framework. This network structure impacts computational efficiency and speed, which are crucial factors for real-time applications [69]. The YOLO model, on the other hand, is a one-stage object section approach that is known for significant speed and real-time performance. This makes it well suited for autonomous mobile robot navigation, in which prompt decision making is important for obstacle avoidance and motion control.

### 3.6. Obstacle Avoidance

Ensuring the safety of the working environment is a primary priority when deploying mobile robots for navigation tasks in complex environments. The safety solution should be able to perceive the environment and take proactive actions to avoid obstacle collisions using reliable sensors [49]. The mobile robot should have the capability to identify a safe and efficient path to navigate within its operational environment, which may contain static and dynamic obstacles to the target destination. Different learning-based obstacle avoidance algorithms have been developed to enable robots to effectively and precisely complete intended tasks. Some are integrated with local and global planners to efficiently adjust the direction and speed of robot motion in response to detected obstacles within static and dynamic environments to generate an improved path to reach the target location [70]. Recent review studies, learning-based models in robotic navigation, have demonstrated notable success by learning and generating obstacle data from environment sensors. These models extract obstacle features from images and video streams, allowing them to classify and locate different obstacles within the given environment. The integration of these models into robot operating system (ROS)-based planners has shown improved performance in robotic navigation. Planning algorithms like the dynamic window approach (DWA) [6] have good capabilities in a dynamic and complex environment and have been widely combined with learning algorithms for more capability, efficiency, and intelligent path planning [71].

### 3.7. Path Planning

Autonomous learning in path planning has made significant progress in recent times, where technologies such as CNN and deep reinforcement learning have been increasingly adopted. Path planning entails a sequence of configurations based on robot types and environment models that enable robots to navigate from a starting point to a target location [72]. The environment can be mapped to represent geometric information about the environment and connectivity between different nodes or maples. The map-based method enables the robotic solutions to compute the robot’s dynamics and environment representation for an optimal global path planning to the goal [73]. For local path planning, it relies on real-time sensory information to navigate safely in the presence of static and dynamic obstacles. Another aspect of path planning configuration is the maples model, which requires no predefined map of the environment but rather capitalises on frameworks like deep learning models to learn and enhance optimal navigation strategies. Path planning in an MRO hangar can be challenging, as the environment is often changing and complex with a high density of obstacles. To ensure a robust obstacle-free path, ongoing research is focusing on path tracking [74], advanced deep learning [75], and hybrid approaches for more autonomous and intelligent robot path planning to target locations [71].

### 3.8. Path Tracking

Safe and efficient robot navigation requires a path tracking system that guides mobile robots along the planned trajectory to a target location, managing and minimising deviation from the planned route. This involves continuous monitoring and updating of the planned route based on sensor feedback and the changing environment. The work in [74] reviewed path tracking algorithms relative to high and low speeds. For high-speed applications, the reaction time available for the robots to perceive, process, and respond to obstacles was significantly reduced at high velocities, making it harder to execute quick and sharp manoeuvres without compromising stability or safety. Looking at low-speed use cases, the application of robotic systems in MRO hangars involved low-speed movement and the requirement for precise path tracking in complex settings. The low-speed movement of these robots led to path tracking errors, especially when dealing with sharp turns and frequent changes in direction. Accurate modelling of low-speed dynamics is essential to adjust the robot’s behaviour for optimal path tracking. The combination of adaptive control systems, sensor technologies [76], and advanced deep learning techniques have been shown to enhance robust real-time path tracking capability for robot navigation in such scenarios. From the study in [77], the most applied path tracking algorithms include pure pursuit (PP) [78], model predictive control (MPC) [79,80], as well as learning-based models to generate control laws leveraging training data and experience from a variety of scenarios [81].

## 4. Learning-Based Navigation Techniques (Methods)

The dynamic and unforeseeable state of edge cases in the real world makes the application of the navigation task challenging. Ensuring that the systems can detect and respond effectively to changing and unstructured scenarios is essential for safe and reliable navigation [32]. The conventional approach, which works best in a static environment, is known to be computationally intensive and must be adjusted to varying environment states and motion dynamics. The learning-based approach has been instrumental in addressing the limitations of traditional methods. These methods utilise complex neural network architecture to process sensory data and extract relevant features, allowing them to adapt to a wide range of environmental structures and make more informed decisions [36]. The primary focus of this paper is on the integration of deep learning methods that specifically address challenges associated with obstacle detection, avoidance, and path planning in environments that are both dynamic and complex.

### 4.1. Deep Reinforcement Learning (DRL)

**Figure 6:**
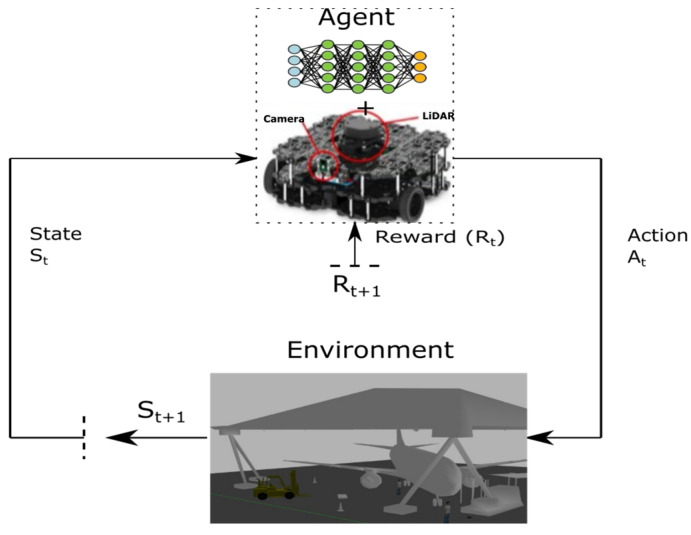
Overview of interaction between agent and environment.

This represents a transformative autonomous navigation approach that combines the perception ability of deep learning and the decision-making potential of reinforcement learning to effectively map sensory input to navigation actions, leading to an improved end-to-end navigation process. The DRL-based navigation model, a description of which is shown in Figure 6, has demonstrated great capacity to achieve safety, adaptability, and efficiency, learning about the workspace with less reliance on the accuracy of sensor information. The deep neural network functionalities of the DRL, like CNN and autoencoder (AE), can automatically extract varying features from highly complex environments. Researchers have adopted this paradigm and have developed different approaches that address autonomous navigation challenges by leveraging DRL algorithms like proximal policy optimisation algorithms (PPOs) [82], deep deterministic policy gradient algorithms (DDPGs) [83], trust region policy optimisation (TRPO) [84], and others. Most of these algorithms are constrained by sparse rewards [85], which impacts model training and convergence rate. Some proposed methods have been shown to discretise the action space; however, these have achieved limited success in addressing the complexity of certain settings. On the other hand, the policy gradient approach, utilising gradient descent, has been applied to solve continuous space problems by calculating the policy parameters to maximise the expected reward [86]. In the context of achieving optimal navigation in complex environments, value-based RL algorithms have shown enhancement for applications involving discrete action spaces by using an argmax to choose the action with the highest Q-value [87]. Further improvement is achieved by proper tuning of system hyperparameters and incorporation of deep learning functions. This includes multiple hidden layers, extensive datasets, ReLU activation functions, and Adam optimiser, among others. Such configurations have been demonstrated to achieve efficiency in obstacle detection, avoidance, and path planning in autonomous navigation systems. Some other methods are highlighted in Table 2, illustrating their specific contributions.

### 4.2. Object Detection Model

Effective obstacle perception and detection are fundamental requirements for ensuring robots can plan collision-free movements. The mobile robot utilises its sensing systems, including LiDARs or cameras, to perceive environmental features that could potentially interfere with navigation tasks. Various methodologies are adopted to improve these capabilities. Some are designed for static settings, detailed environment maps, sensor fusion, and dynamic scenarios, with the potential to improve navigation ability. A major advancement in this field has been the incorporation of machine learning into object detection systems, allowing for the creation of more flexible and effective systems. This learning-based method drives learning from data to perform MR navigation tasks through domain knowledge [44]. In the MRO hangar context, the changing environmental structure with varying object types, occlusion, and low-light conditions limits the capability of the learning model and requires more training costs to scale to new environment variables. These are among the major difficulties of obstacle detection in real-world mobile robot navigation that have resulted in many environment-related accidents. Object detection algorithms use the explicit definition of environment variables like obstacle size, shape, depth, and object distance range in the environment [55,57] to predict obstacles, improve obstacle avoidance, and plan a smooth route to the destination. Additionally, Table 3 highlights ROS deep learning solutions that have been developed and can be applicable based on their capabilities. Most of these algorithms employ pretrained object detection networks to develop models relevant to obstacle avoidance and pathfinding optimisation. Among the widely employed object detection algorithms utilised in robotics and other domains include CNN, Faster R-CNN, and YOLO.

#### 4.2.1. CNN

CNN represents a deep learning architecture composed of multiple convolutional layers. This method demonstrates the capacity to automatically discover and extract important elements from images, facilitating object recognition and classification [55]. Numerous researchers have used this method to develop models for object detection and collision avoidance. For instance, Qi et al. [93] developed a modern CNN technique to identify and classify obstacles in complex environments; they highlighted the improvements in obstacle identification. Additionally, Mechal et al. [94] presented a CNN model trained with different types of images, such as RGBD, RGB and HSD, enabling the classification of obstacle avoidance actions. The authors of [93,95] used CNN to estimate the depth of objects in an image to consequently steer commands for a quadrotor. This study provided evidence demonstrating the superior performances of CNNs compared with traditional methods. Similarly, Liu et al. [96] proposed a CNN vision-based model for obstacle avoidance, aiming to generate steering commands for a mobile robot while reducing the need for complex and time-consuming hand-engineering of features. The ability of CNN to learn feature representations directly from raw image data without the need for manual feature extraction marks a significant advancement in the field of object detection and has led to widespread adoption in various applications, including autonomous vehicles and robotics.

#### 4.2.2. Faster R-CNN

Faster R-CNN is an extension of CNN based on the region proposal network (RPN), which is classified as a two-stage convolutional neural network. This sophisticated approach is intended to create bounding boxes for each proposed region and then extract important features from these regions, making it easier to classify the objects contained in each region later [69]. Faster R-CNN is mentioned in Lee et al.’s [97] paper, where it was used to assist unmanned vehicles to avoid collision. Mahendrakar et al. [69] employed this technique to ease robot navigation while showing the excellent accuracy of the Fast R-CNN. In the study conducted by Hakim et al. [98], Faster R-CNN was explained to operate in two phases. Initially, it generates bounding boxes around several objects within an image, and subsequently, in the second phase, the proposed regions underwent classification to accurately detect objects. Compared with other object detection models like YOLO, Faster R-CNN is more precise in the object detection process with a more comprehensive and multiple-layer structure [93], and this makes it an attractive option for real-time applications. However, the gain in accuracy comes at the expense of real-time identification speed, as the algorithms exhibit slower performance in this regard. In addition, significant time is required to collect training data in the case of multiple-layer CNN models [99]. Real-world applications require obstacle detection systems with high accuracy and real-time capabilities to efficiently respond to the increasingly complex demands of different domains. Faster R-CNN is potentially more computationally intensive and of low inference.

#### 4.2.3. YOLO

YOLO, in contrast, uses a single layer of object recognition and simultaneously creates bounding boxes around objects using a grid-based methodology. This is achieved using a technology that divides images into grid cells and then creates bounding boxes that include the recognised items [69]. To address map-building challenges, Emmi et al. [100] conducted a comparative analysis of YOLOv3 with Retina Net-Resnet, ultimately selecting YOLO as the preferred solution. Cao et al. [101] relied on YOLOv3 to build their detection system with high accuracy and processing speed to enable efficient underwater robot navigation. Additionally, Mahendrakar et al. [69] used YOLOv5 in autonomous navigation to show the efficiency of YOLO in real-time situations. In the context of real-time object detection, the speed of the process holds significant importance for effective obstacle avoidance. Comparative analysis between YOLO, CNN, and Faster R-CNN [102] highlights YOLO’s superior adaptability in real-time object detection scenarios. While YOLO’s speed and adaptability make it an attractive option for real-time applications, its dependency on extensive training data underscores a potential limitation, especially in an environment with data scarcity. Therefore, the choice between object detection models should be guided by specific application requirements to balance factors such as speed and accuracy.

**Table 3:** Recent solution with integrated ROS and object detection models for robot navigation.

Ref.	Sensor	Environment	Learning Algorithm	Strength	Limitation
[103]	Vision sensor	Dynamic in sim/real world	YOLOv4 and ROS DWA	Increased detection speed of 15 FPS higher	3% reduced detection accuracy
[104]	Vision sensors	Static and dynamic in simulation	Yolov3 and ROS DWA	Improved obstacle avoidance with about 82% detection accuracy	Default ROS planners used require improvement
[105]	Monocular camera	Complex in simulation	Fully connected network (FCN) and A*	Reduced path length and optimised trajectory	The model cannot adapt to changes due to limited data coverage
[106]	RGBD cameras	Static in simulation	CNN and ROS	Improved autonomous navigation with camera and IMU sensors	Limited in low-light situations
[107]	LiDAR and camera	Complex in simulation	ROS and YOLOV3	Improve obstacle detection and navigation performance	Limited to a static environment
[108]	RGBD image	Static in real world	Yolo3 and odometry programme	Successful collision-free trajectory	Require trade-off between speed and obstacle detection accuracy
[109]	LiDAR	Static in real world	YOLO + simultaneous localisation and mapping (SLAM)	70% less time to compute inference, which improved obstacle detection and navigation	Used a sensor with limited functionality and in a less complex environment
[110]	Monocular camera	Dynamic real-world	MASK R CNN (region convolutional neural network)	Improved obstacle detection in low-light situations	Obstacle detection accuracy is limited when the robot to obstacle distance is less than 1.5 m
[111]	LIDAR	Static in real world	Yolov3 + ROS global planner	Improved obstacle avoidance in a static environment	Robots lose the path to the target location when two obstacles are close to each other

### 4.3. Hybridisation with Neural Network

Hybridisation of algorithms has become commonplace, as it integrates the strength of learning and nonlearning methods together to achieve more effective, accurate, and reliable obstacle detection and avoidance solutions. This section investigates notable publications on classic ROS-based and machine learning methods. The concept of robot operating system (ROS) navigation planners provides a detailed framework that can integrate different types of algorithms and sensors to develop complex robotics applications [112]. Emerging solutions have utilised neural networks (NNs) to optimise traditional path planning and obstacle avoidance techniques in recent times [43,113]. A neural network is a layered framework of interconnected nodes that takes input based on a designed task and produces network prediction or classification as output. The authors [113] applied a long short-term memory (LSTM) neural network using robot pose and agent to obstacle distance as the input to solve end-to-end path planning while avoiding a dynamic obstacle. Other authors applied multilayer perceptron (MLP) network classic path planning algorithms to demonstrate improved performance [114]. The authors of [115] used a padding mean neural dynamic model to address the challenges of traditional neural dynamics by enhancing the completeness and optimality properties of path planning in static and dynamic environments. More algorithms and their performance capabilities are discussed and compared in Table 4. The integration of multiple techniques can increase computational demand and the complexity of the system and affect real-time performance. Other factors, like environment dependency, may affect generalizability to other platforms.

## 5. Drawbacks and Future Work

Most obstacle detection, avoidance, and path planning solutions are unstable and must be robust to be applicable in real-world scenarios. These systems rely on various parameters, and finding suitable configurations is difficult and results in unpredictable performance [113]. This can be exacerbated when dealing with limited training data. Generating data and experience from the robot’s environment through perception and interaction can be challenging owing to environmental factors, system functionalities, and even the robot’s dynamics. These can impact the functionality and capability of the algorithm, especially in cases in which sufficient and relevant data are required for effective robot training. Conversely, recent research has demonstrated the significance of extensive and versatile data and experience in improving the end-to-end robot navigation experience. Robots tend to generalise better in this case and can apply learned skills to novel situations, making them reliable in real-world applications.

The effectiveness of learning-based methods also depends on the quality of sensor information. Poor sensor data can affect the stability, reliability, and performance of robotic solutions. They can be subject to high levels of inaccuracies and noise in sensor data that breed uncertainties and deviations in performance, which affects safe navigation [117]. Calibrating and simulating sensors to match real sensor specifications are essential to bridge the gap. Also, augmenting simulated sensor data to replicate the noise, uncertainty, and limitations of real-world sensor properties should be able to fine tune the robot’s ability accordingly. Combining data from multiple sensors with sensor fusion techniques has been proven to bridge the reality gap.

Another major research concern is the difficulty in transferring the trained model from simulation to real-world robot platforms. The simulation environment accommodates high-level collisions and reduces training difficulties compared with training in a physical environment, which can be dangerous and cause damage to the robot [114]. For the learning-based algorithm to be helpful for robot navigation, it must be swift in adapting to the new environment and generalising to related tasks without the cost of additional training. Tai et al. [118] demonstrated a maples motion planner built on observations from a low-dimensional range laser sensor and asynchronous deep-RL technique to generalise to a real differential robot platform without further retraining. Achieving efficient transfer without performance degradation for obstacle avoidance and route planning solutions requires developing a high-fidelity simulation environment that closely mimics the real-world environment [96]. However, not all tasks can leverage knowledge from another; some necessitate specialised models tailored to specific challenges. For example, in an MRO hangar, the mobile robot domain experience might be specific to obstacles unique to aviation maintenance settings, and this complexity requires a customised model. However, the integration of advanced neural networks and deep learning techniques has marked a significant improvement in the field of intelligent robotics, paving the way for more efficient and reliable solutions.

## 6. Conclusions

This research review provides a comprehensive overview of the current landscape of learning-based object detection and avoidance in emerging intelligent and autonomous vehicles. The complex and dynamic MRO hangar environment requires robust and intelligent robot navigation systems to manage tasks with high demand efficiently. To achieve this, an optimal path planning model is required to generate an obstacle-free and shortest route to the target point through efficient obstacle detection and avoidance solutions. Recent work in this area showed improvement in safety and reliability and also a significant contribution to broader AI-driven robotics in dynamic real-world applications. We critically examined the inherent functionalities and challenges associated with applying these models in AV environments, particularly focusing on the trade-offs between detection accuracy and avoidance efficiency. Our exploration of various learning-based techniques has underscored their potential to significantly utilise extensive datasets and experience learning to enhance adaptability and task generalisation, factors vital for real-time robot navigation in a changing MRO hangar environment. The choice of models for safe and efficient robot navigation is a requirement and operational environment-specific. Relative to the MRO hangar scenario, the combination of deep learning architecture and advanced path planning and obstacle avoidance strategies from our recent work using LiDAR and camera data fusion have been shown to enhance reliability and adaptability in changing structures.

The findings showed that integrating an object detection model into a navigation system enhances the obstacle detection rate by 20–30% over conventional methods. YOLO models showed excellent results in most review papers and were recommended as the best fit for obstacle detection. Also, the learning-based methods contributed to a reduction in path planning computation time and path length by 10–15%. These results significantly reduce the incidence of navigation errors and improve the overall safety and reliability of the navigation process. However, considering complexities and uncertainties in the MRO hangar, the research recommends the development of domain-specific trained models. These are models that are trained with data collected specifically from different MRO hangar environments to improve the robustness and generalizability necessary for real-time and real-world operations. The performance of these systems should be continuously monitored and evaluated to maintain high safety standards in changing real-world conditions. There is still a need for continued research in this domain, particularly in developing algorithms that balance performance with computational demands. These advancements promise to make robotics systems more adaptive, intelligent, and efficient in the complex environment.

## Figures and Tables

**Table 1:** Comparing traditional and intelligent robotics applications in MRO hangar.

Task	Traditional Approach	Intelligent Robotics Application
Inspection accuracy	Dependent on programme quality and human interaction	Enhanced accuracy facilitated by learning and data analysis
Automation level	Manual–semiautomated with human oversight	Fully automated with limited human input
Algorithms	Predefined algorithms combined with basic sensor input	Sensor fusion, advanced navigation algorithms, and machine learning models
Obstacle detection and navigation	Basic, simple path planning algorithms	Using real-time and advanced deep learning models to enhance path planning
Task performance	Best suited for repetitive and defined tasks	Able to manage varied and complex tasks
Cost	Higher cost for longer maintenance time and error management	Lower maintenance engagements and cost

**Table 2:** Comparison of papers on DRL-based robot navigation systems.

Ref.	Principle	Method	Model	Strength	Limitation
[88]	Avoid obstacles and navigation	Deep Q network (DQN) and duelling double deep Q network (D3QN)	Model-free	Can learn from very depth noise	Applicable in less complex settings
[89]	Path planning in continuous space	DDPG	Model-free	Optimal path generation with less environmental information	Predefined tracks can obstruct efficient navigation in highly dynamic scenes
[83]	Generalisation ability of path planning	LSTM + DDPG	Model-free	100% success rate, 18.8% better training time and 21% shorter distance covered	Only considered static obstacles
[90]	Collision-free navigation	DDPG with a separate experience	Model-free	An improved replay mechanism was adopted for training and improved network performance	Network parameters are randomly set and limited in real-time situations
[91]	Object recognition and robot navigation	Deep neural network (DNN)	Map-based	Average recognition accuracy of 80%	Require a more complex environment to validate real-world applicability
[92]	Object detection and tracking for navigation	CNN	Model-based	Can detect and track multiple obstacles	The default ROS algorithms used are limited in complex and real-time demanding environments

**Table 4:** Strengths and limitations of hybrid systems solutions in obstacle avoidance and path planning.

Ref.	Model Composition	Strength	Limitation
[114]	Multilayer perceptron (MLP)	Avoid obstacles and find the shortest path significantly	Inability to integrate with other path planers
[113]	Long short-term memory (LSTM) synchronised with ROS	Completeness and optimality of path planning	The experiment is limited to the simulation environment
[96]	CNN-based ROS with ROS-cafe	Improved obstacle avoidance and motion control	Limited in dynamic and complex environments
[105]	FCN and A*	Obstacle avoidance and path planning	Use of only a camera, which limits the field of view (FOV)
[106]	CNN and ROS	Obstacle detection and avoidance	Limited in a dynamic setting
[116]	Sampling-based planner (RRT*) and conditional variational autoencoder (CVAE)	Improved path planning	Scaling to complex problems can be difficult
